# Non-fluoroscopic vs. fluoroscopic radiofrequency catheter ablation for pediatric atrioventricular nodal reentrant tachycardia: a comparative study of procedural characteristics and ablation site

**DOI:** 10.3389/fcvm.2025.1602458

**Published:** 2025-07-10

**Authors:** Chieh-Mao Chuang, Pi-Chang Lee, I-Hsin Tai, Ying-Hsuan Peng, Wen-Po Fan, Yu-Shin Lee, Ming-Chih Lin, Sheng-Ling Jan, Yun-Ching Fu, Shih-Ann Chen

**Affiliations:** ^1^ Division of Pediatric Cardiology, Children’s Medical Center, Taichung Veterans General Hospital, Taichung, Taiwan; ^2^ Division of Cardiology, Department of Internal Medicine, Asia University Hospital, Taichung, Taiwan; ^3^ Department of Cardiology, China Medical University Children’s Hospital, Taichung, Taiwan; ^4^ Department of Pediatrics, College of Medicine, China Medical University, Taichung, Taiwan; ^5^ Division of Pediatric Cardiology, Department of Pediatrics, Chung Shan Medical University Hospital, Taichung, Taiwan; ^6^ Division of Pediatric Cardiology, Department of Pediatrics, Taipei Veterans General Hospital, Taipei, Taiwan; ^7^ Division of Pediatric Cardiology, Department of Pediatrics, Chang Gung Memorial Hospital, Taoyuan, Taiwan; ^8^ Department of Post-Baccalaureate Medicine, College of Medicine, National Chung Hsing University, Taichung, Taiwan; ^9^ Cardiovascular Center, Taichung Veterans General Hospital, Taichung, Taiwan; ^10^ Institute of Clinical Medicine, and Cardiovascular Research Center, National Yang Ming Chiao Tung University, Taipei, Taiwan; ^11^ Division of Cardiovascular Medicine, Department of Medicine, China Medical University Hospital, China Medical University, Taichung, Taiwan

**Keywords:** radiofrequency ablation, atrioventricular nodal reentrant tachycardia, pediatric, non-fluoroscopic, catheter ablation

## Abstract

**Background:**

Non-fluoroscopic cryoablation is considered safe for pediatric atrioventricular nodal reentrant tachycardia (AVNRT), but concerns about prolonged procedures and recurrence persist, requiring continued use of radiofrequency ablation (RFA). Non-fluoroscopic RFA, guided by three-dimensional mapping, offers enhanced precision. This study compares its safety and effectiveness with fluoroscopic RFA in pediatric AVNRT.

**Methods:**

We retrospectively analyzed children undergoing RFA without (X− group) or with (X+ group) fluoroscopy at multiple centers (2011–2024). Patients who received fluoroscopic and three-dimensional mapping guidance were excluded. Baseline characteristics, electrophysiological data, and ablation outcomes were compared.

**Results:**

Among 119 patients (X+, 57; X−, 62), there was a significantly higher rate of successful ablation sites in the lower Koch triangle on 3D electroanatomical mapping (3D-EAM). However, the procedure time, acute success rate, recurrence-free survival of AVNRT, and injury to the conduction system were similar between the groups. No permanent complete atrioventricular block was observed in either group. The longer procedure time was independently correlated with ablation location outside of the low Koch triangle, lower common pathway block, and slow pathway modification. Dual AV nodes without inducible tachycardia had lower AVNRT-free survival than typical and atypical AVNRT (80% vs. 96.5% vs. 100%, *p* = 0.025). Younger children can achieve successful ablation with fewer ablation pulses and a smaller ablation catheter profile, with similar recurrence and conduction system injury.

**Conclusions:**

Non-fluoroscopic RFA, guided by 3D-EAM, achieves comparable acute and mid-term outcomes to fluoroscopic RFA in pediatric AVNRT. The successful site of slow pathway ablation was significantly lower when using 3D-EAM compared with fluoroscopy.

## 1 Introduction

Radiofrequency ablation (RFA) is used to treat patients with atrioventricular reentrant tachycardia (AVNRT). A meta-analysis revealed that RFA is associated with a high acute success rate, short procedure time, low complication rate, high long-term success rate, and low long-term recurrence rate (1). Although the procedure has a low complication rate, complete AV block requiring permanent pacemaker implantation occurs in a small proportion of patients. In children, the small area of the Koch triangle and the thinness of the tissue that covers the AV node make ablation for AVNRT challenging. More generator changes are required, and more pacemaker-related complications occur in pediatric procedures due to the longer life expectancy of children. Cryoablation was developed to safely treat AVNRT while avoiding the complication of AV block, which requires pacemaker implantation. However, compared with RFA, cryoablation has a lower long-term success rate, a higher recurrence rate, and a longer procedure time (1). Three-dimensional (3D) mapping systems have been used in ablation for AVNRT to reduce or eliminate the use of fluoroscopy and allow more precise 3D navigation. The use of 3D navigation has enabled non-fluoroscopic cryoablation for AVNRT (2–4). However, non-fluoroscopic RFA for pediatric AVNRT has not been extensively studied (5). Whether non-fluoroscopic RFA is safer and more effective than fluoroscopic RFA in pediatric AVNRT remains unknown.

We compared the safety and medium-term outcomes of non-fluoroscopic and fluoroscopic RFA in pediatric AVNRT.

## 2 Methods

This study was carried out at two tertiary medical centers. A retrospective database was established, and patient data were reviewed after institutional review board approval.

### 2.1 Patients

From December 2011 to January 2024, 126 patients aged <19 years with AVNRT received slow pathway (SP) RFA. The X+ group comprised 57 consecutive patients (from December 2011 to November 2018) who received SP RFA with fluoroscopic guidance, without 3D electroanatomical mapping (3D-EAM) assistance. The X− group consisted of 62 consecutive patients (from March 2019 to January 2024) who received SP RFA guided by 3D-EAM without fluoroscopy. Seven patients who received SP RFA were excluded due to the use of 3D-EAM and fluoroscopy. The clinical and demographic data collected included sex, age, height, weight, body surface area, clinical manifestations, and medical history. Informed consent was obtained from all patients, and the study protocol complied with the ethical guidelines of the Helsinki Declaration of 1975 and obtained prior approval by the Institutional Review Board of Taichung Veterans General Hospital (TCVGH-IRB No. CE22018A-2).

### 2.2 Electrophysiologic study

Every patient discontinued antiarrhythmic drugs (AADs) for at least five half-lives prior to electrophysiologic study (EPS). A 3D mapping system (EnSite NavX, Abbott, MN, USA) was used in the X− group. An EPS was performed, and the SP was determined through sustained SP conduction with burst pacing or prolongation of the atrial–His (AH) or ventriculoatrial (VA) interval from ≥50 ms in 10 ms decrements during extrastimulation. Tachycardia was induced, with or without isoproterenol infusion. Its mechanism was confirmed as AVNRT through pacing maneuvers. AVNRT was categorized as slow–fast, fast–slow, or slow–slow on the basis of the VA interval (60 ms) and AH/HA ratio. Patients with inducible AVNRT who did not meet the AH or VA jump criteria were classified as having a continuous AV node function curve.

### 2.3 Radiofrequency ablation

**Figure 1:**
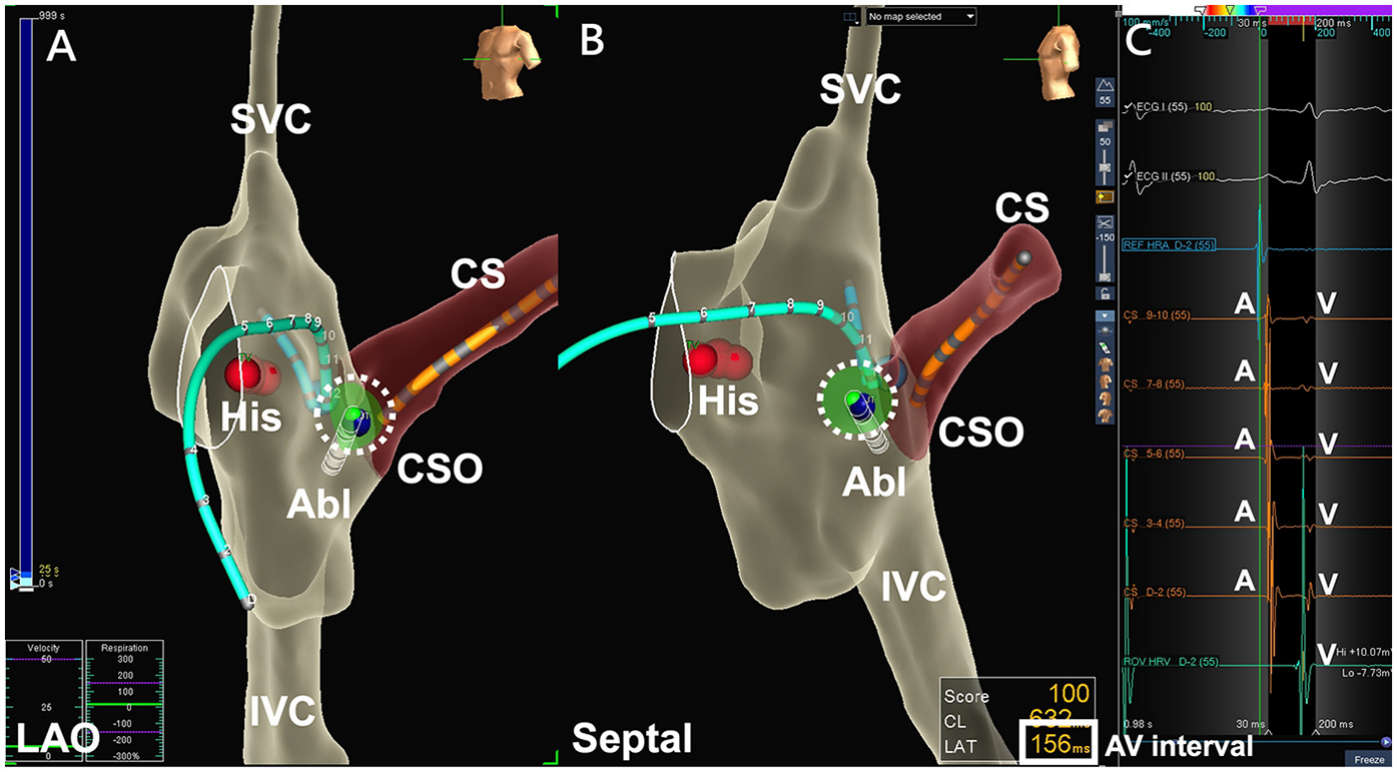
Non-fluoroscopic AVNRT ablation by a 3D-EAM system. **(A)** RF ablation over the low Koch region on a LAO projection by the 3D-EAM system, with the red dots indicating the His cloud. **(B)** A clearer spatial relationship between the ablation catheter and the Koch triangle was found when using septal projection. The catheter contact can be revealed from either local electrograms or the projection point area of the ablation catheter tip (dotted circle). **(C)** Monitoring of the real-time AV interval (bolded square) can be achieved by using the appropriate window of the 3D-EAM system during ablation. LAO, left anterior oblique; His, His bundle; CSO, coronary sinus ostium; CS, coronary sinus; SVC, superior vena cava; IVC, inferior vena cava; Abl, ablation catheter; A, atrial electrogram; V, ventricular electrogram; AV interval, atrioventricular interval.

A 5.5 or 7 Fr non-irrigated RFA catheter (Ablaze Fantasista, Japan Lifeline, Tokyo, Japan; Livewire, Abbott, MN, USA) was used to map the lower part of the Koch triangle via femoral access. The ideal ablation location was one with an AV ratio of 1:2–1:5 with a “bump-and-spike” atrial electrogram. For the fluoroscopic approach, the positions of the coronary sinus (CS) ostium and His bundle were identified through real-time biplane fluoroscopy [right anterior oblique (RAO) and left anterior oblique (LAO) views]. In the X− group, the positions of the CS ostium and His bundle were confirmed using the CS catheter and His cloud marking in the 3D mapping system (LAO and lateral views). Then we began ablation below the CS roof using a local electrogram of slow potential and a tip temperature of 50°C, with the power limited to 50 W. If the target temperature was not achieved, the power limit was titrated up to 55 W. No additional mitigating strategies were employed to control or modulate the target temperature. If an accelerated junctional rhythm was detected, 60 s of RFA was performed with atrial pacing. If more than two additional instances of consecutive accelerated junctional beats or AH prolongation occurred during ablation, the operator stopped the ablation using a foot pedal. The real-time AV interval was also monitored during ablation in the X− group using the 3D mapping system (Figure 1). After 60 s of RFA, the presence and function of the SP were evaluated. A booster RFA of 30–60 s was applied if SP was not present or there was only one AV nodal echo beat. Another EPS with isoproterenol infusion was performed after ablation. Acute success of ablation for AVNRT was defined as an outcome of either non-inducible AVNRT without evidence of AH or VA jump (SP elimination) or a maximum of one AV nodal echo beat during isoproterenol infusion (SP modification). After ablation, all patients received isoproterenol for the inducibility test.

### 2.4 Follow-up

Outpatient follow-up visits for ECG occurred 1, 6, 12, 18, 24, and 30 months after ablation. Recurrence was defined as the recurrence of clinical symptoms with a documented abnormal heart rate or ECG indicating supraventricular tachycardia (SVT).

### 2.5 Statistical analysis

Excel 365 (Microsoft, Redmond, WA, USA) and SPSS version 19.0 (SPSS, Chicago, IL, USA) were used to perform data analysis. Continuous variables are presented as medians with interquartile ranges (IQRs), and categorical variables are presented as absolute and relative frequencies. For group comparisons, we used the Mann–Whitney *U* test or Kruskal–Wallis test for continuous variables and the chi-square test (or Fisher's exact test) for nominal variables. The Kaplan–Meier method was used to estimate the overall recurrence rate of AVNRT after ablation. The log-rank test was performed to compare the recurrence rates between groups. The scatter plot between procedure time and the time sequence of the ablation between the two groups was analyzed using Spearman's rho correlation. Univariate and multivariate logistic regression models were used to assess the risk factors for a prolonged procedure time. Odds ratios (OR) and 95% confidence intervals (CI) were calculated for each model. A two-tailed *p*-value of <0.05 indicated significance.

## 3 Results

### 3.1 Baseline characteristics

Among the 119 study patients, the median age was 13.7 years. Of the patients, 62 were in the X− group and 57 were in the X+ group. The baseline characteristics of the patients are presented in Table 1. Two patients had previous AVNRT ablation with recurrence, and three patients had congenital heart disease (Ebstein's anomaly, ventricular septal defect, and L-transposition of the great arteries), all of whom were in the X+ group. All demographic data were comparable between the groups.

**Table 1:** Demographic data.

Variable	Total (*n* = 119)		X− (*n* = 62)		X+ (*n* = 57)		*p*-value
Age (years)	13.7	(12.1–15.9)	13.5	(12.5–15.7)	13.7	(11.7–16.2)	0.882
Gender							0.960
Female	54	(45.4%)	28	(45.2%)	26	(45.6%)	
Male	65	(54.6%)	34	(54.8%)	31	(54.4%)	
Body weight (kg)	50.0	(40–64)	50.1	(39.6–63.4)	50.0	(45–65.3)	0.600
Body height (cm)	157.5	(151–169.6)	158.0	(152.3–169.7)	157.0	(150.5–169.5)	0.710
BSA (m^2^)	1.5	(1.3–1.7)	1.5	(1.3–1.7)	1.5	(1.3–1.7)	0.803
Congenital heart disease	3	(2.5%)	0	(0%)	3	(5.3%)	0.107
Previous AVNRT ablation	2	(1.7%)	0	(0%)	2	(3.5%)	0.227
Antiarrhythmic drug	49	(41.2%)	21	(33.9%)	28	(49.1%)	0.091
Clinically documented tachycardia	95	(79.8%)	47	(75.8%)	48	(84.2%)	0.254

Mann–Whitney *U* test, chi-square test, Fisher's exact test.

X–, non-fluoroscopic; X+, fluoroscopic; BSA, body surface area; AVNRT, atrioventricular nodal reentrant tachycardia.

*p < 0.05.

**p < 0.01.

### 3.2 Electrophysiological characteristics

The electrophysiological characteristics of the groups are shown in Table 2. EPS revealed that most of the patients had inducible AVNRT (90.8%), most of which was slow–fast AVNRT (87.4%). Some patients had AVNRT with multiple AV nodal pathways (10.9%), which was higher in the X– group. The most common type of SP was antegrade conduction (61.3%). A lower common pathway block was experienced by 14.9% of the patients. A significantly higher inducible concurrent arrhythmia was observed during EPS in the X– group (37.1% vs. 15.8%), the most common type being atrial fibrillation or atrial flutter. Continuous AV node function curve was more common in the X+ group than in the X− group (43.9% vs. 22.6%, *p* = 0.013). Before ablation, 27% of patients presented with sustained AVNRT without isoproterenol, 57.4% had sustained AVNRT with isoproterenol, 10.4% revealed non-sustained AVNRT with isoproterenol, and 5.2% showed non-inducible AVNRT with isoproterenol.

**Table 2:** Electrophysiology characteristics.

Variable	Total (*n* = 119)		X− (*n* = 62)		X+ (*n* = 57)		*p*-value
Pre-ablation inducibility	108	(90.8%)	57	(91.9%)	51	(89.5%)	0.643
Slow–fast AVRNT	104	(87.4%)	55	(88.7%)	49	(86.0%)	0.652
Fast–slow AVNRT	8	(6.7%)	7	(11.3%)	1	(1.8%)	0.063
Slow–slow AVNRT	5	(4.2%)	3	(4.8%)	2	(3.5%)	>0.99
Multiple forms of AVNRT	5	(4.2%)	4	(6.5%)	1	(1.8%)	0.367
No. of slow pathways							0.003**
Single	106	(89.1%)	50	(80.6%)	56	(98.2%)	
Double	12	(10.1%)	11	(17.7%)	1	(1.8%)	
Triple	1	(0.8%)	1	(1.6%)	0	(0%)	
Slow pathway direction							1.000
Antegrade	73	(61.3%)	38	(61.3%)	35	(61.4%)	
Retrograde	3	(2.5%)	2	(3.2%)	1	(1.8%)	
Bidirectional	43	(36.1%)	22	(35.5%)	21	(36.8%)	
Lower common pathway block	14	(14.9%)	8	(12.9%)	6	(18.8%)	0.544
Continuous AV node curve	39	(32.8%)	14	(22.6%)	25	(43.9%)	0.013*
Other tachycardia	32	(26.9%)	23	(37.1%)	9	(15.8%)	0.009**
AF or AFL	25	(78.1%)	19	(82.6%)	6	(66.7%)	0.370
Atrial tachycardia	1	(3.1%)	1	(4.3%)	0	(0%)	1.000
Accessory pathway	5	(15.6%)	4	(17.4%)	1	(11.1%)	1.000
FV bypass tract	2	(6.3%)	0	(0%)	2	(22.2%)	0.073

Chi-square test, Fisher's exact test.

X–, non-fluoroscopic; X+, fluoroscopic; AVNRT, atrioventricular nodal reentrant tachycardia; AV, atrioventricular; AF, atrial fibrillation; AFL, atrial flutter; FV, fasiculoventricular. Slow–fast, fast–slow, slow–slow, and multiform AVNRT are independent variables.

* *p* < 0.05.

**p < 0.01.

### 3.3 Procedural characteristics and clinical outcomes

**Figure 2:**
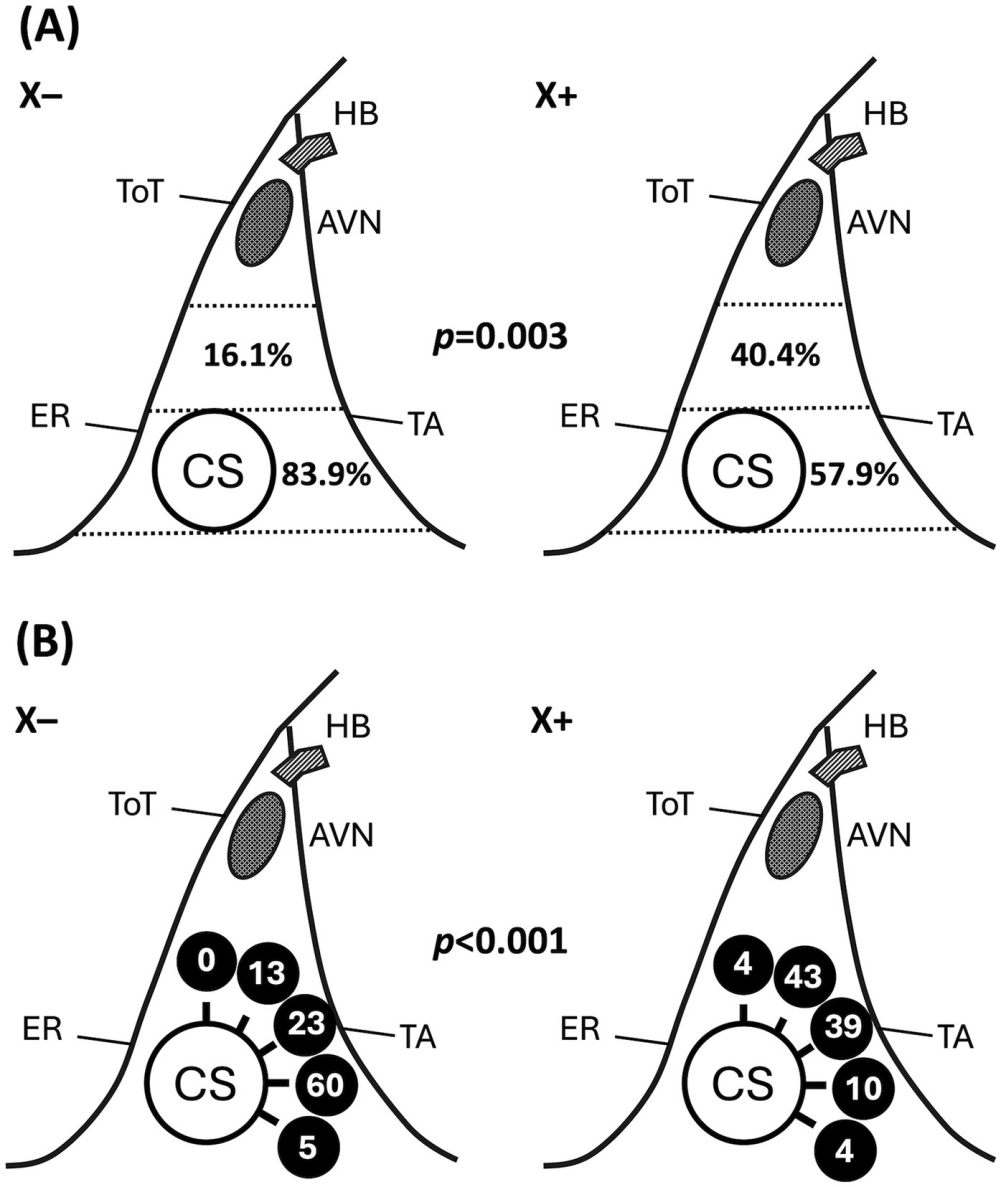
Ablation site distribution between non-fluoroscopic and fluoroscopic RFA. **(A)** Significant higher successful ablation site in the fluoroscopic RFA group, categorized by low, middle, and high Koch triangle. **(B)** Significant lower successful ablation site in the non-fluoroscopic RFA group according to the coronary sinus viewed as a clock face. X− = non-fluoroscopic approach. X+ = fluoroscopic approach. ToT, tendon of Todaro; ER, eustachian ridge; CS, coronary sinus; HB, His bundle; AVN, atrioventricular node; TA, tricuspid annulus.

**Figure 3:**
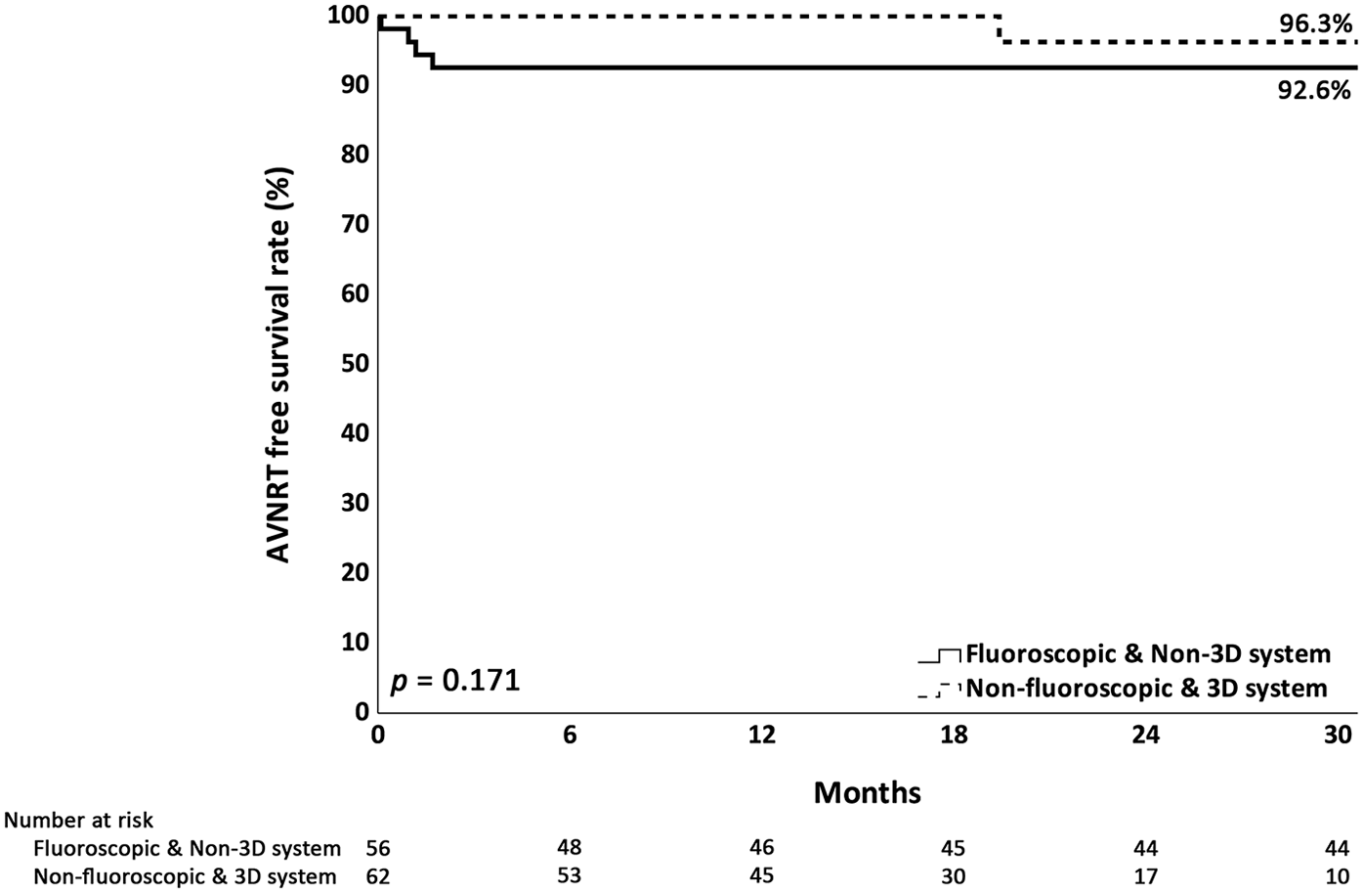
Kaplan–Meier analysis of AVNRT-free survival. Kaplan–Meier analysis showed no significant differences in AVNRT-free survival when comparing X− and X+ groups. X− = non-fluoroscopic approach. X+ = fluoroscopic approach.

The procedural characteristics and clinical outcomes of the groups are presented in Table 3. The 3D-EAM system was used on all patients in the X− group, but was not used for any of the patients in the X+ group. A small portion (7.8%) of AVNRT ablation was performed with a 5.5 Fr ablation catheter, rather than a 7 Fr non-irrigated ablation catheter. No successful RF applications were delivered within the coronary sinus or on the left atrial side. All ablation lesions were confined to the right atrium. The most common location of the SP was in the lower third of the Koch triangle (70.6%), followed by the middle third of the Koch triangle (27.7%). Notably, a higher proportion of middle Koch ablation was observed in the X+ group (40.4% vs. 16.1%). If we classified the successful ablation site in terms of the coronary sinus viewed as a clock face, there was a significant difference between the groups, with the most common site over 3 o'clock in the X– group and 1 o'clock in the X+ group (Figure 2). Slightly more SP modification (52.5%) than SP elimination (47.5%) was performed. In 118 of 119 cases (99.2%), acute success was achieved, with the only failed case in the X+ group. The procedure time was similar between the groups. The fluoroscopy time was significantly longer in the X+ group than that in the X− group (23 vs. 0 min). No major complications occurred in either group, but minor complications occurred in seven cases that were comparable between the groups. Six of these seven cases involved permanent injury to the AV conduction system that did not require pacemaker implantation, and one case involved a femoral hematoma. Injury to the AV conduction system occurred in 14 cases (11.8%), of which 8 involved transient AV block during the procedure and spontaneous recovery. All eight cases with transient AV block demonstrated complete recovery of AV nodal conduction, either immediately after cessation of RF energy delivery or before the procedure concluded. Among them, two experienced transient third-degree AV block, three had transient high-degree AV block, and three had transient first-degree AV block. These occurred in five patients from the X– group and three from the X+ group. Three cases involved a permanent first-degree AV block (two in the X+ group and one in the X− group). In the X− group, two cases involved the right bundle branch block due to the manipulation of recording catheters, and one involved a second-degree Mobitz Type 1 AV block. A treadmill exercise test was performed in the case of the second-degree AV block. The patient recovered AV conduction during exercise and was asymptomatic. Because the X+ group was a historical control group, the average duration of follow-up was significantly longer than that of the X− group (30 vs. 17.4 months, *p* < 0.001). The recurrence rate was higher in the X+ group than that in the X− group, without reaching statistical significance (4.2% vs. 1.6%, *p* = 0.189). Recurrence-free survival did not differ significantly between the groups (*p* = 0.171; Figure 3).

**Table 3:** Procedural characteristics and results.

Variable	Total (*n* = 119)		X− (*n* = 62)		X+ (*n* = 57)		*p*-value
3D system	62	(52.1%)	62	(100%)	0	(0%)	
EnSite	62	(100%)	62	(100%)	0	(0%)	
Abl catheter size (Fr), *n* (%) (*n* = 116)							1.000
5.5	9	(7.8%)	5	(8.1%)	4	(7.4%)	
7	107	(92.2%)	57	(91.9%)	50	(92.6%)	
Slow pathway location							0.003**
Low Koch	85	(71.4%)	52	(83.9%)	33	(57.9%)	
Middle Koch	33	(27.7%)	10	(16.1%)	23	(40.4%)	
Not found	1	(0.8%)	0	(0%)	1	(1.8%)	
Successful site with respect to the coronary sinus (o'clock), *n* (%) (*n* = 111)							<0.001**
12 o'clock	2	(1.8%)	0	(0%)	2	(4.1%)	
1 o'clock	29	(26.1%)	8	(12.9%)	21	(42.9%)	
2 o'clock	33	(29.7%)	14	(22.6%)	19	(38.8%)	
3 o'clock	42	(37.8%)	37	(59.7%)	5	(10.2%)	
4 o'clock	5	(4.5%)	3	(4.8%)	2	(4.1%)	
Slow pathway treatment result (*n* = 118)							0.371
Elimination	56	(47.5%)	27	(43.5%)	29	(51.8%)	
Modification	62	(52.5%)	35	(56.5%)	27	(48.2%)	
Acute success	118	(99.2%)	62	(100%)	56	(98.2%)	0.479
Procedure time (min)	105.0	(85–135)	105.5	(90.8–145.5)	100.0	(85–132)	0.222
Fluoroscopic time (min)	0		23.0	(16.3–32.8)			
Minor complication	7	(5.9%)	5	(8.1%)	2	(3.5%)	0.442
Conduction system injury							0.654
Transient AV block	8	(6.7%)	5	(8.1%)	3	(5.3%)	
First-degree AV block	3	(2.5%)	1	(1.6%)	2	(3.5%)	
Second-degree Mobitz Type 1 AV block	1	(0.8%)	1	(1.6%)	0	(0%)	
Right bundle branch block	2	(1.7%)	2	(3.2%)	0	(0%)	
Duration of follow-up (months)	29.3	(12.8–30)	17.4	(11.2–25)	30.0	(30–30)	<0.001**
AVNRT recurrence	5	(4.2%)	1	(1.6%)	4	(7.1%)	0.189

Mann–Whitney *U* test, chi-square test, Fisher's exact test.

X–, non-fluoroscopic; X+, fluoroscopic; AV, atrioventricular; AVNRT, atrioventricular nodal reentrant tachycardia.

* *p* < 0.05.

** *p* < 0.01.

### 3.4 Procedure time and learning curve

Patients were divided into two groups based on the third quartile of the procedure time (135 min). Univariate analysis by logistic regression showed that higher body height, the presence of a lower common pathway block, ablation outside the low Koch region, and slow pathway modification were associated with a longer procedure time (Table 4). Independent factors for prolonged procedure time were the presence of a lower common pathway block and ablation outside the Koch region by multivariate analysis.

**Table 4:** Risk factors for procedure time >135 min.

Variable	Univariate model			Multivariate model (*n* = 117)			Multivariate model (*n* = 92)		
	OR	(95% CI)	*p*-value	OR	(95% CI)	*p*-value	OR	(95% CI)	*p*-value
Age (year)	1.11	(0.95–1.29)	0.189						
Body weight (kg)	1.02	(1.00–1.04)	0.090						
Body height (cm)	1.03	(1.00–1.06)	0.044*	1.03	(1.00–1.06)	0.097			
Lower common pathway block	3.44	(1.07–11.12)	0.039*				5.95	(1.46–24.17)	0.013*
Fluoroscopy use									
X+	1.00								
X−	2.08	(0.87–4.96)	0.100						
Slow pathway location									
Lower Koch	1.00			1.00					
Non-lower Koch	2.85	(1.17–6.93)	0.021*	2.56	(1.01–6.48)	0.047*	4.87	(1.57–15.12)	0.006**
AVNRT type									
Fast–slow	1.04	(0.20–5.44)	0.966						
Slow–slow	2.15	(0.34–13.53)	0.416						
Multiple type AVNRT	2.15	(0.34–13.53)	0.416						
Slow pathway treatment result									
Elimination	1.00			1.00					
Modification	2.49	(1.02–6.06)	0.045*	2.25	(0.89–5.70)	0.088	3.52	(1.16–10.64)	0.026*

Logistic regression.

Fr., French.

* *p* < 0.05.

** *p* < 0.01.

**Figure 4:**
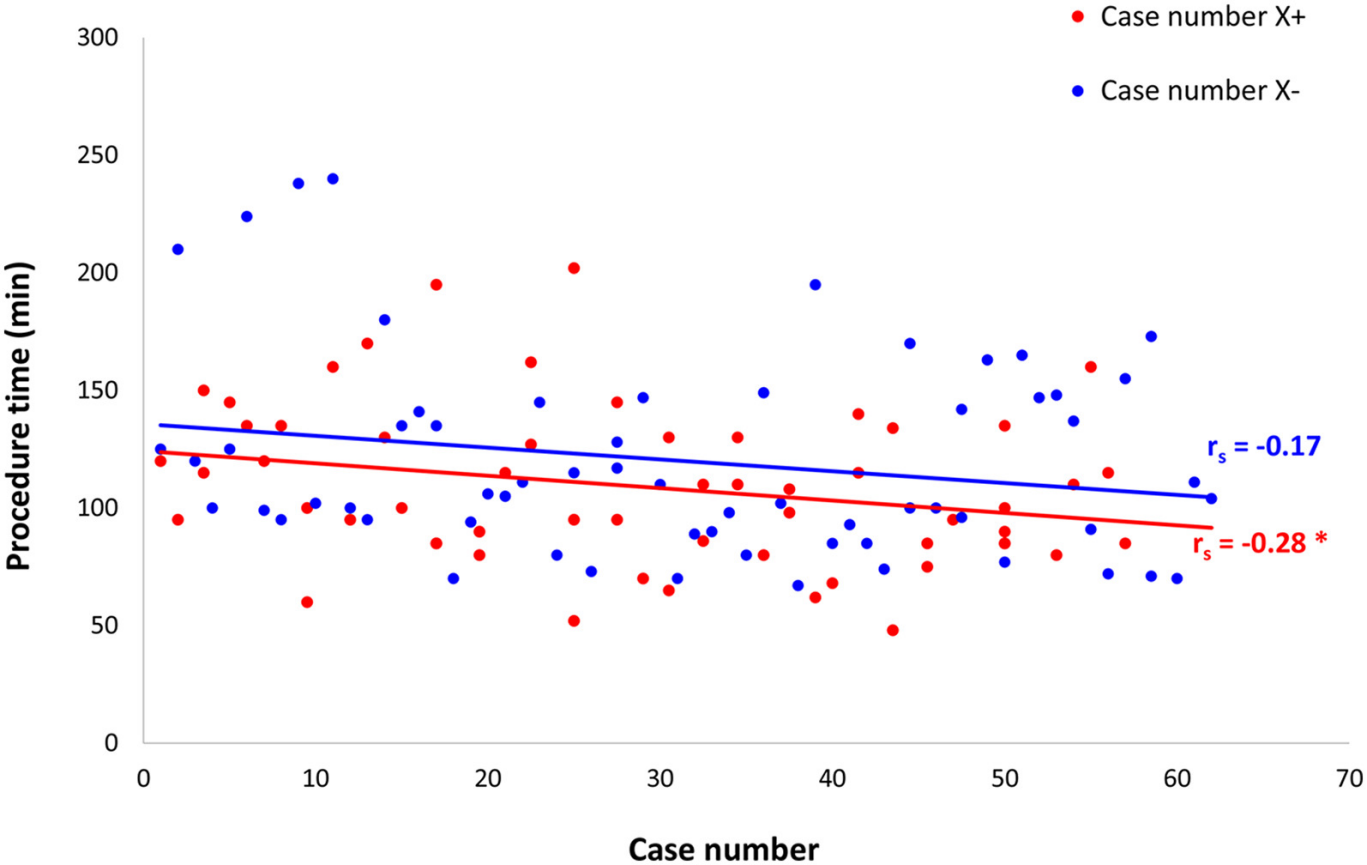
Scatter plot for procedure time vs. rank of the case by the time of operation. The scatter plot demonstrates decreased procedure time when performing more procedures in both groups; however, only the X+ group reached statistical significance. *p < 0.05.

In both groups, there was a trend of decreasing procedure time as the number of procedures increased (Figure 4 and Supplementary Table S1); however, only the X+ group reached statistical significance.

### 3.5 Different types of AVNRT

**Figure 5:**
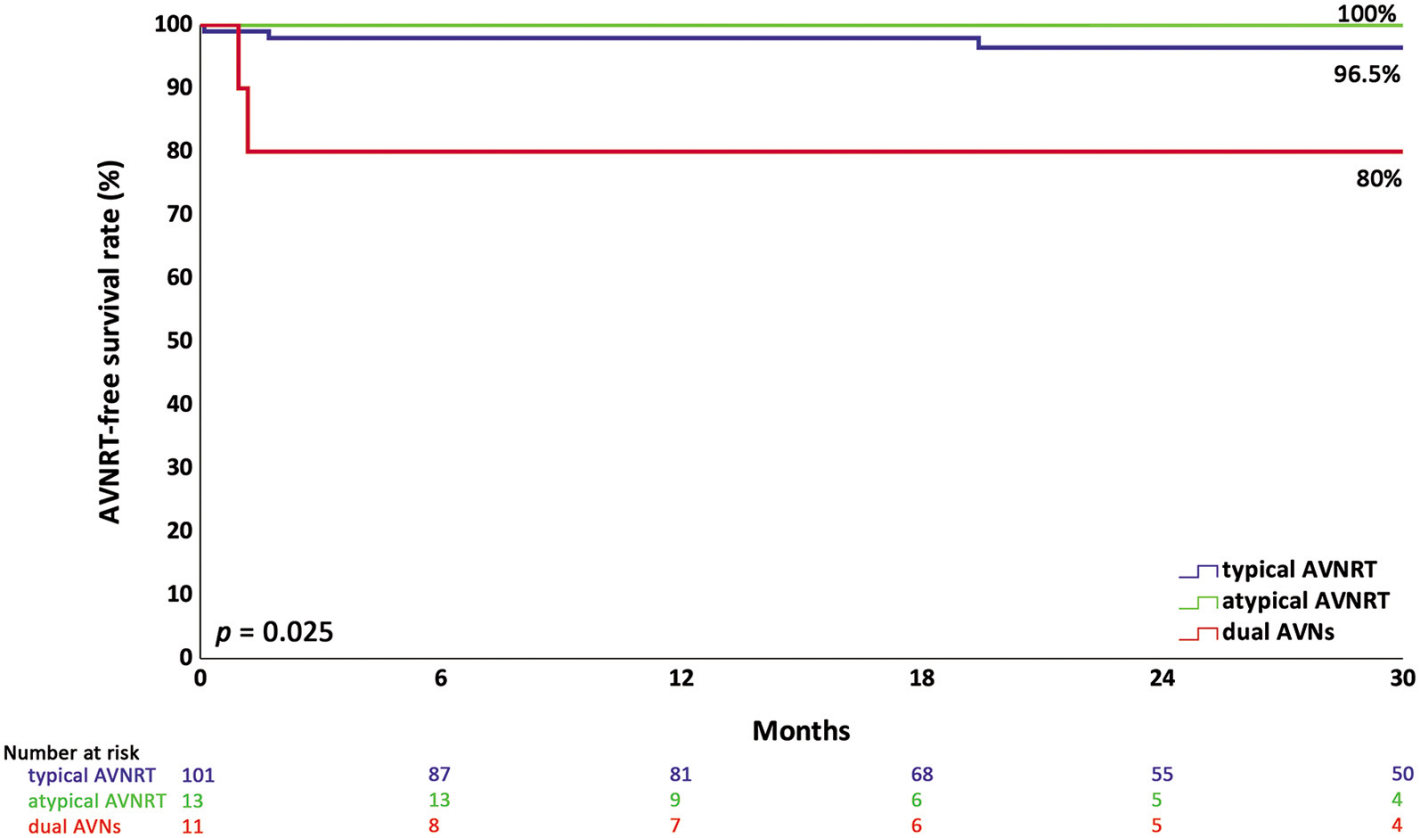
Kaplan–Meier analysis of AVNRT-free survival in different types of AVNRT. Kaplan–Meier analysis showed significantly lower AVNRT-free survival in children with dual AVNs without inducible AVNRT receiving RFA. AVNRT, atrioventricular nodal reentrant tachycardia; dual AVNs, dual atrioventricular nodes without inducible AVNRT.

The patients were grouped into three types, which were typical AVNRT, atypical AVNRT, and dual AV nodal physiology without inducible AVNRT (dual AVNs). The last type had clinically documented paroxysmal supraventricular tachycardia. The demographic and procedural data comparing different types of AVNRT are shown in Supplementary Table S2. Most of the cases were typical AVNRT, followed by atypical AVNRT and dual AVNs. There were no differences in demographic and EPS data, except for a twofold greater AAD use and fewer concurrent other tachycardias in the dual atrioventricular node (AVN) group. The ablation characteristics and clinical outcomes were comparable between the groups, except for a trend of a higher rate of recurrence at 30 months of follow-up in the dual AVN group (18.2%). Significantly lower AVNRT-free survival was observed in the dual AVN group compared with other groups (80% vs. 96.5% vs. 100%, *p* = 0.025) (Figure 5). Pairwise comparison of AVNRT-free survival between groups showed that the only significant difference was between dual AVNs and typical AVNRT (*p* = 0.014).

The cohort included 13 cases of atypical AVNRT, of which 6 exhibited multiform patterns. Fast–slow AVNRT was observed in eight patients and slow–slow AVNRT in six patients; notably, one patient exhibited all three forms—fast–slow, slow–slow, and slow–fast AVNRT. Compared with typical AVNRT, this group tended to have longer procedure times, a higher proportion of successful ablation sites in the low Koch triangle region, and a lower recurrence rate. However, these differences did not reach statistical significance due to the limited sample size.

### 3.6 RF ablation for children of younger age

We defined younger children by age in the first quartile (≤12.1 years) and compared them with older children whose ages ranged from the second to the fourth quartiles. Demographic and procedural data for these groups are shown in Supplementary Table S3. The median age of the two groups was 9.9 and 14.8 years. Significantly lower body weight was observed in younger children (33 vs. 56 kg). More AADs were used in younger children (58.1% vs. 36.8%). There was more inducible AVNRT without isoproterenol in younger children, and more patients needed isoproterenol to induce AVNRT in older children. A greater use of 5.5 Fr ablation catheters was observed in younger children. Significantly fewer ablation pulses were found in younger children. Procedure time, recurrent rate, acute success, complication, and location of the ablation were comparable between the groups.

## 4 Discussion

### 4.1 Summary

To our knowledge, this is the first study to compare non-fluoroscopic and fluoroscopic RFA for pediatric AVNRT. The main findings are comparable procedure times, acute success rates, complication rates, and recurrence rates. The successful site was significantly lower in the Koch triangle based on 3D-EAM compared with results achieved using fluoroscopy. The presence of a lower common pathway block and ablation outside of the low Koch region were independent risk factors associated with long procedure times. Furthermore, the procedure time decreased significantly with greater numbers of patients treated in the X+ group, but not in the X− group. In different types of AVNRT, dual AVNs only without inducible AVNRT had significantly lower survival without AVNRT. In different age groups, younger children had a procedure time, non-fluoroscopic procedure, complication rate, and recurrence rate similar to those of older children, except for fewer RF ablation pulses.

### 4.2 Acute outcomes of ablation for pediatric AVNRT

AVNRT treatment through the RFA of the SP region was first studied in 1992 (6). Since then, SP RFA has been the mainstay treatment for AVNRT due to its high acute success rate, low complication rate, and low recurrence rate. Although complications are rare, AV block that requires permanent pacemaker implantation is a potential and serious complication. Due to this safety concern, SP cryoablation has been researched since 2000 (7). The use of cryoablation in pediatric AVNRT is increasing due to the high risk of AV block with RFA and the long life expectancy of children after AV block. Although permanent AV block after cryoablation has not been reported, meta-analyses have identified longer procedure times and higher recurrence rates than those associated with RFA (1, 8). An analysis of a large multicenter pediatric ablation registry for Wolff–Parkinson–White syndrome revealed that using 3D-EAM for catheter ablation significantly reduced fluoroscopy time and improved the acute procedural success rate (9). By using the 3D-EAM system, the ablation time can be reduced without lengthening the procedure time or increasing the acute complication rate, according to several meta-analyses (10–12). We used a 3D-EAM system for non-fluoroscopic RFA for pediatric AVNRT and observed procedure times, acute success rate, and acute complication rates comparable to those achieved by fluoroscopic ablation.

### 4.3 Long-term results of ablation for pediatric AVNRT

Two key long-term outcomes of ablation for AVNRT are recurrence and permanent AV block. Three meta-analyses of mixed arrhythmia studies have indicated no significant differences in arrhythmia recurrence or complications between 3D-EAM and conventional procedures (10–12). In a large single-center study of catheter ablation for SVT in adults, the use of conventional fluoroscopic ablation was correlated with significantly increased recurrence (hazard ratio, 3.03) and late complications (advanced AV block requiring pacemaker implantation) (13). Few retrospective single-center studies of pediatric AVNRT receiving RFA have used a 3D-EAM system to reduce reliance on fluoroscopy use (5, 14–16). One study reported AV block in three patients after RFA, two of whom had complex congenital heart disease; the other was a 15-month-old child (16). However, three other studies using RFA with 3D-EAM guidance to treat pediatric AVNRT reported no AV block. The rate of AV block appears lower when a 3D-EAM system is used rather than fluoroscopic guidance (17–21). In our study, no permanent AV block requiring pacemaker implantation occurred in the X− group, which had a low recurrence rate (2.9%) after a median follow-up of 1.5 years.

Cryoablation remains widely used in pediatric AVNRT ablation due to the anatomical constraints of the smaller Koch triangle. In the largest comparative cohort of pediatric AVNRT patients undergoing cryoablation (*n* = 1,201) vs. RF ablation (*n* = 1,046), redo ablation rates were 2.5% vs. 0.9%, respectively, with mean time to recurrence of 203 vs. 315 days (22). In comparison, our RF-only cohort (*n* = 119) had a higher redo ablation rate of 4.2% and a shorter time to recurrence, with a median duration of 36 days. This difference may be attributed to our smaller sample size and the longer study period, which may have introduced temporal and observational bias.

### 4.4 Non-fluoroscopic ablation for pediatric AVNRT

Fluoroscopy is the key to cardiac catheterization. However, the ionizing radiation released during fluoroscopy exerts deterministic and stochastic effects on exposed medical personnel and patients. Although deterministic effects, such as skin injury or cataracts, only occur above a certain radiation threshold, the risk of stochastic effects increases linearly with the dose; that is, any amount of radiation increases the risk of cancer. Radiation exposure should be reduced to as low as reasonably achievable to avoid stochastic effects. Using a 3D-EAM and adequate radiation protection during fluoroscopy can almost completely eliminate the risk of deterministic effects, but only forgoing the use of fluoroscopy can prevent stochastic effects. Due to the anatomical approach of AVNRT RFA, whether the Koch triangle using 3D-EAM is the same as that obtained using fluoroscopy becomes a crucial issue during the non-fluoroscopic approach. In the present study, successful ablation sites were significantly lower on the 3D-EAM compared with fluoroscopy. This discrepancy regarding successful sites using different image modalities can be explained by the imprecise location of the coronary sinus on fluoroscopy. Three studies have investigated non-fluoroscopic ablation for pediatric AVNRT: Two used cryoablation (2, 3), and one case series comprising both adults and children used RFA (5). Furthermore, all three were single-arm studies without control groups. Our study is the first case series on pediatric AVNRT treated with non-fluoroscopic RFA.

### 4.5 Non-fluoroscopic ablation learning curve

In our study, the procedure time decreased significantly with the number of procedures completed in the X+ group; this trend was also observed in the X− group without statistical significance. Two small pediatric case series on non-fluoroscopic ablation of SVT did not demonstrate any effects of a learning curve on procedure time (22, 23), but one large multicenter adult registry analysis identified a significant shortening of procedure time after non-fluoroscopic ablation for SVT, which was performed in 60 cases (23). One small case series of non-fluoroscopic ablation for AVNRT used cryoablation and demonstrated a significant reduction in procedure time as the number of completed procedures increased (3). However, another small series using fluoroscopic RFA for pediatric AVNRT observed no significant differences between early and more recent procedures (14). Thus, for AVNRT non-fluoroscopic RFA, there are far more technical challenges compared with fluoroscopic RFA, resulting in only a significant trend of reduction in procedure time in the X+ group after more procedures had been completed.

### 4.6 Risk factors related to the prolonged procedure time

In our cohort, the presence of a lower common pathway block and ablation outside of the low Koch region were independent risk factors associated with prolonged procedure time. As a result of the slow pathway ablation performed according to the anatomical approach, the low Koch region was always the first site of attempt. If we did not achieve successful slow pathway ablation over the low Koch region, ablation outside of the low Koch region would be the second anatomical target. This explains why the prolonged procedure occurred more frequently when ablating outside of the low Koch region. However, it is difficult to explain why the presence of a lower common pathway block during AVNRT was a risk factor for prolonged procedure. According to the results of the largest study of RFA in pediatric AVNRT, a significantly longer procedure time was observed in older children and atypical AVNRT, without logistic regression analysis (24). The present investigation is the first study to use logistic regression analysis in order to identify independent risk factors for prolonged procedure time in pediatric AVNRT receiving RFA.

### 4.7 RFA for different types of AVNRT with diverse recurrence-free survival

Dual AVNs only without inducible AVNRT had the lowest recurrence-free survival after RFA compared with typical and atypical AVNRT in our study. O'Leary et al. (24) found that dual AVNs only and atypical AVNRT were two independent risk factors for higher recurrence. However, atypical AVNRT had a similar recurrence-free survival to typical AVNRT in our study.

### 4.8 Efficacy and safety of RFA for AVNRT in younger children

In our cohort, RFA for AVNRT in young children had fewer RF lesions and RF time and no complications compared with older children. There was a similar recurrence of AVNRT between younger and older children in our cohort. O'Leary et al. (24) also showed a significantly shorter RF time and lesions with a significantly shorter procedure time in younger children with AVNRT receiving RFA. No differences in AVNRT recurrence were observed between younger and older children in their study. Despite the smaller area of the Koch triangle in younger children, RFA not only did not carry a higher risk of AV block, but also shortened the RF time and reduced RF lesions in both studies.

### 4.9 Study limitations

First, this was a retrospective analysis of multicenter cohort data. Second, the duration of follow-up was significantly longer in the X+ group than that in the X− group, because the X+ group was a historical control group. Third, due to the small number of cases in both groups, the rates of recurrence and complication may not represent the general population.

## 5 Conclusions

The use of the 3D-EAM for non-fluoroscopic RFA targeting of the lower area of the Koch triangle can produce outcomes similar to those of fluoroscopic RFA targeting a higher position for pediatric AVNRT. Non-fluoroscopic SP RFA is safe and effective in the medium term for pediatric patients.

## Data Availability

The original contributions presented in the study are included in the article/Supplementary Material, further inquiries can be directed to the corresponding author.
