# Supplementary material for: Non-fluoroscopic vs. fluoroscopic radiofrequency catheter ablation for pediatric atrioventricular nodal reentrant tachycardia: a comparative study of procedural characteristics and ablation site
Source: Front Cardiovasc Med. 2025 Jul 10;12:1602458. doi: 10.3389/fcvm.2025.1602458 (PMC12287064; doi:10.3389/fcvm.2025.1602458)
Supplement: Supplementary file 1 [file Datasheet1.pdf]

Supplementary Table 1. Correlation of procedure time (min) and case number

	n	r	*p* value
Case number			
X+	57	-0.28	0.033*
X-	62	-0.17	0.189

Spearman's rho. * $p < 0.05$. X–, nonfluoroscopic; X+, fluoroscopic.

Supplementary Table 2. Characteristics of different AVNRT subtypes

	Typical AVNRT (n=102)		Atypical AVNRT (n=13)		Dual AVNs (n=11)		*p* value
	Median	IQR	Median	IQR	Median	IQR	
Age (year)	13.7	(12-15.8)	13.5	(12.6-16.1)	14.3	(10-16.2)	0.901
Body weight (kg)	51.0	(39.7-64.3)	49.1	(38.2-60)	51.5	(45.5-65)	0.693
Gender, n (%)							0.329
Female	50	(49.0%)	5	(38.5%)	3	(27.3%)	
Antiarrhythmic drug, n (%)	38	(37.3%)	6	(46.2%)	9	(81.8%)	0.017*
Clinically documented tachycardia, n (%)	82	(80.4%)	9	(69.2%)	11	(100%)	0.147
No. of slow pathway, n (%)							0.054
Single	93	(91.2%)	10	(76.9%)	9	(81.8%)	
Double	9	(8.8%)	3	(23.1%)	1	(9.1%)	
Triple	0	(0%)	0	(0%)	1	(9.1%)	
Slow pathway location, n (%)							0.318

Lower Koch	71	(69.6%)	10	(76.9%)	8	(72.7%)	
Middle Koch	30	(29.4%)	2	(15.4%)	3	(27.3%)	
Lower and middle Koch	0	(0%)	1	(7.7%)	0	(0%)	
Not found	1	(1.0%)	0	(0%)	0	(0%)	
Procedure time (min)	105.0	(85.8-135.5)	135.0	(88-177.5)	110.0	(95-135)	0.278
Nonfluoroscopic ablation	49	(48.0%)	8	(61.5%)	5	(45.5%)	0.635
Acute success, n (%)	101	(99.0%)	13	(100%)	11	(100%)	1.000
Slow pathway treatment, n (%)							0.586
Elimination	47	(46.5%)	8	(61.5%)	5	(45.5%)	
Modification	54	(53.5%)	5	(38.5%)	6	(54.5%)	
Minor complication, n (%)	6	(5.9%)	0	(0%)	1	(9.1%)	0.574
Recurrence, n (%)	3	(3.0%)	0	(0%)	2	(18.2%)	0.087

Kruskal-Wallis test. Fisher's exact test.*$p<0.05$, **$p<0.01$.

Supplementary Table 3. Characteristics of the younger (age≤12.1 years old) and older pediatric groups

	Age of Q1 (n=31)		Age of Q2-4 (n=95)		*p* value
	Median	IQR	Median	IQR	
Age (year)	9.9	(8.2-11.5)	14.8	(13.3-16.4)	<0.001**
Body weight (kg)	33.0	(26.6-38.8)	56.0	(47-66.9)	<0.001**
Gender, n (%)					0.911
Female	14	(45.2%)	44	(46.3%)	
Antiarrhythmic drug, n (%)	18	(58.1%)	35	(36.8%)	0.038*

3D system, n (%)	15	(48.4%)	54	(56.8%)	0.411
Pre-ablation inducibility type, n (%)					0.021*
Sustained AVNRT without ISO	14	(46.7%)	19	(20.7%)	
Sustained AVNRT with ISO	11	(36.7%)	60	(65.2%)	
Non-sustained AVNRT with/without ISO	3	(10.0%)	9	(9.8%)	
Non-inducible AVNRT	2	(6.7%)	4	(4.3%)	
Slow pathway location, n (%)					0.577
Lower Koch	25	(80.6%)	64	(67.4%)	
Middle Koch	6	(19.4%)	29	(30.5%)	
Lower and middle Koch	0	(0%)	1	(1.1%)	
Not found	0	(0%)	1	(1.1%)	
Ablation catheter size, n (%)					<0.001**
5.5 Fr.	8	(26.7%)	2	(2.2%)	
7 Fr.	22	(73.3%)	91	(97.8%)	
Acute success, n (%)	31	(100%)	94	(98.9%)	1.000
Slow pathway treatment, n (%)					0.960
Elimination	15	(48.4%)	45	(47.9%)	
Modification	16	(51.6%)	49	(52.1%)	
Nonfluoroscopic ablation	12	(38.7%)	50	(52.6%)	0.178
Procedure time (min)	100.0	(85-135)	110.0	(91-145)	0.304
Ablation application	6.0	(4-20)	15.0	(5-37.5)	0.036*
Ablation time (sec)	169.5	(128.3-282.8)	254.5	(153.3-417)	0.055
Minor complication, n (%)	0	(0%)	7	(7.4%)	0.192

Conduction system injury, n (%)					0.179
No AVB	25	(80.6%)	86	(90.5%)	
Transient	4	(12.9%)	5	(5.3%)	
1st degree AVB	2	(6.5%)	1	(1.1%)	
2nd degree Mobitz type 1	0	(0%)	1	(1.1%)	
RBBB	0	(0%)	2	(2.1%)	
Recurrence at 30 months, n (%)	2	(6.5%)	3	(3.2%)	0.597
Duration of follow-up (months)	29.3	(12.7-30)	30.0	(14.1-30)	0.853

Mann-Whitney U test. Fisher's exact test. Chi-square test. *p<0.05, **p<0.01. Q1, first quartile; Q2-4, second to fourth quartiles. Fr., French.
